# Alcohol Toxicity in the Developing Cerebellum

**DOI:** 10.3390/diagnostics14131415

**Published:** 2024-07-02

**Authors:** Hiroshi Mitoma, Mario Manto, Aasef G. Shaikh

**Affiliations:** 1 Medical Education Promotion Center, Tokyo Medical University, Tokyo 160-0023, Japan; 2 Unité des Ataxies Cérébelleuses, Service de Neurologie, CHU-Charleroi, 6000 Charleroi, Belgium; mario.manto@ulb.be; 3 Service des Neurosciences, University of Mons, 7000 Mons, Belgium; 4 Louis Stokes Cleveland VA Medical Center, University Hospitals Cleveland Medical Center, Cleveland, OH 44106, USA; axs848@case.edu

**Keywords:** alcohol, ethanol, cerebellum, cerebellar ataxias, fetal alcohol spectrum disorder, internal model, functional connectivity

## Abstract

The impact of ethanol on the fetus is a significant concern as an estimated 2–5% of live births may be affected by prenatal alcohol exposure. This exposure can lead to various functional and structural abnormalities in the cerebral cortex, basal ganglia, diencephalon, and cerebellum, resulting in region-specific symptoms. The deficits relate to the motor and cognitive domains, affecting, in particular, general intelligence, attention, executive functions, language, memory, visual perception, and social skills—collectively called the fetal alcohol spectrum disorder (FASD). Recent studies suggest that damage to the developing cerebellum (in form of alcohol exposure) can impair the cortical targets of the cerebello-thalamo-cortical tract. This malfunction in the cerebello-cerebral loop optimization may be due to disruptions in the formation of the foundational elements of the internal model within the developing cerebellum. Alcohol exposure targets multiple nodes in the reciprocal loops between the cerebellum and cerebral cortex. Here, we examine the possibility that prenatal alcohol exposure damages the developing cerebellum and disrupts the connectivity within the cerebello-cerebral neuronal circuits, exacerbating FASD-related cortical dysfunctions. We propose that malfunctions between cerebellar internal model (critically involved in predictions) and cerebral regions contribute to the deficits observed in FASD. Given the major role of the cerebellum in motor, cognitive, and affective functions, we suggest that therapies should target these malfunctions to mitigate the burden of FASD. We discuss the concept of therapies oriented towards malfunctioning cerebello-cerebral loops (TOMCCLs), emphasizing anti-inflammatory strategies and treatments aimed at modulating cerebellar myelination to restore optimal and predictive cerebello-cerebral functions.

## 1. Introduction

### 1.1. Cellular and Molecular Mechanisms Underlying Alcohol-Induced Functional Disorders and Degeneration

The cerebellum is particularly vulnerable to alcohol toxicity. Alcohol induces functional dysregulation and the cell death of cerebellar neurons, which explain the diverse neurological signs and symptoms of both acute and chronic alcohol toxicity [1,2]. The acute and transient effects are grouped under the term alcoholic cerebellar ataxia [3] or ethanol-induced cerebellar ataxia [4], which is characterized by gait imbalance, a prominent sway in the anteroposterior direction, and scanning speech. These clinical features are due to two alcohol-induced synaptic dysfunctions: (1) distortion of the cerebellar cortex activity by impairing synaptic transmission between Golgi cells and granule cells and (2) disorganization of the outputs from the cerebellar cortex due to the effects of deficits in adenosine-induced inhibition on synaptic transmissions, such as between parallel fibers and Purkinje cells (PCs), parallel fibers (PFs) and basket cells, and basket cells and PCs [2]. 

**Figure 1:**
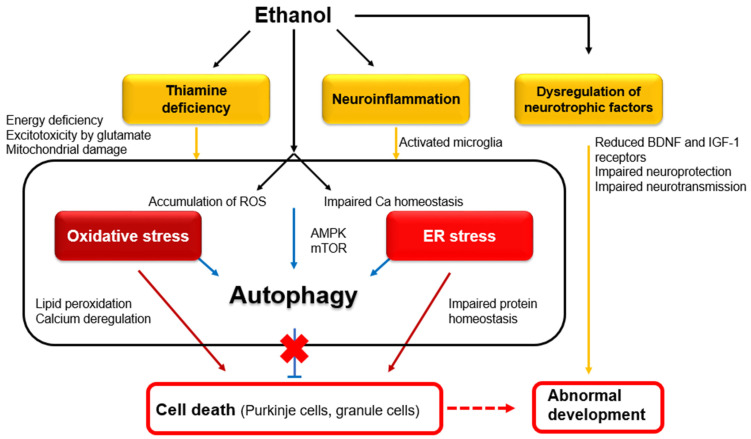
Intracellular mechanisms underlying ethanol-induced neuronal death in adults and abnormal development. Abbreviations: BDNF—brain-derived neurotrophic factor; ROS—reactive oxygen species; AMPK—AMP-activated protein kinase; mTOR—mammalian target of rapamycin [2].

On the other hand, chronic alcohol consumption causes cerebellar atrophy, involving mainly the anterior superior vermis, leading to posture/gait instability [3,5] and cognitive dysfunction, especially executive skills in the Category Test [6]. Maternal exposure to alcohol induces fetal alcohol spectrum disorder (FASD), which is characterized by permanent congenital deficits in both the motor and cognitive domains. FASD is a major source of concern, as ethanol exposure to the developing fetus occurs in 2–5% of live births, and the rate of FASD is estimated at 24 to 48 per 1000 children [7,8]. Some of the clinical features of FASD have a cerebellar nature, including motor incoordination and deficits in sustained attention, verbal fluency, visual perception, and social skills [9,10,11,12]. Notably, the chronic effects of alcohol are induced through common molecular and cellular mechanisms, including neuronal degeneration in adults and FASD during development [2]. In particular, oxidative stress and endoplasmic reticulum (ER) stress represent the primary pro-apoptotic mechanisms [13]. In the context of FASD, oxidative stress and ER stress are intertwined with neuroinflammation [13]. Furthermore, the dysregulation of neurotrophic factors impacts neural development [13]. The relative positioning of these molecular mechanisms in adult degeneration and FASD is shown in Figure 1.

### 1.2. Effects of Alcohol on the Developing Cerebellum

Despite significant advancements in our comprehension of the cellular and molecular mechanisms underlying alcohol-induced functional disorders and cell death, the impacts of alcohol on the developing cerebellum remain insufficiently explored at the level of neural circuits. The reasons why prenatal alcohol exposure leads to persistent disabilities in the motor and cognitive domains remain unclear, especially from the angle of the numerous cerebello-cerebral interactions. 

Notably, the cerebellum plays a critical role in neurodevelopmental disorders, and damage to the developing cerebellum can cause permanent and diverse cognitive dysfunctions [14]. It is generally accepted that deficits in the developing cerebellum can lead to the development of autism spectrum disorder (ASD) and attention-deficit-hyperactivity disorder (ADHD) through the reciprocal cerebro-cerebellar networks [14]. 

Although exposure to alcohol causes injury to a large area (including reductions and abnormalities in the overall brain size and morphology) [15], the location(s) of the core region(s) responsible for FASD profiles have not been mapped yet. However, the analogy between ASD and ADHD suggests a possible scenario: the developing cerebellum is highly vulnerable to the toxic effects of ethanol, and such an impairment exacerbates cerebral cortex dysfunctions by acting on the multiple loops between the cerebellum and cerebral cortex. 

This article aims to examine the pathophysiological roles of the cerebellum in the development of FASD, considering the perspective of internal models, which is the leading theory of cerebellar function. We performed a PubMed search using the keywords/terms “fetal alcohol spectrum disorder” AND “review” AND “2019–2023”. From the identified 168 articles, we checked the abstract and extracted 12 relevant articles [16,17,18,19,20,21,22,23,24,25,26,27]. This article proceeds in four steps: (1) based on the literature search, we summarize the pathological and clinical profiles of FASD; (2) we review the general principles regarding the roles of the cerebellum in neurodevelopmental disorders. We propose that the inability to establish the foundational elements of the internal forward model within the developing cerebellum contributes to the impairment of optimization cerebello-cerebral circuits. (3) We examine the validity of the hypothesis that maternal exposure to alcohol during pregnancy damages the developing cerebellum of the fetus, which leads to a disorganization of the functional neural circuitry between the cerebrum and cerebellum and exacerbation of cerebral dysfunctions, leading to the clinically overt features of FASD. (4) Building upon this hypothesis, we propose a therapeutic approach addressing the malfunction of the cerebello-cerebral loops in individuals affected by FASD.

## 2. Clinical Profiles of FASD

Almost 50 years ago, Jones and Smith [28] identified alcohol as a teratogen. Since then, a wide range of neurobehavioral deficits associated with prenatal alcohol exposure have been collectively categorized under the FASD entity [16,17,18,19,20,21,22,23,24,25,26,27,29]. However, the diagnosis of FASD may be challenging mainly due to a vague and obscure history of prenatal alcohol exposure [16,25,30]. Furthermore, the neuropsychological symptoms of FASD overlap with those of other etiologies [20], and FASD shows wide variability in neuropsychological manifestations [21]. Interestingly, the core symptoms are not seen in the early postnatal period [19]. One study conducted in four US communities between 2010 and 2016 reported the prevalence of FASD to be between 11.3 and 50.0 per 1000 first-grade children (a conservative estimate) [8,31], although this rate is considered to be an underestimate [27].

### 2.1. Pathology

Prenatal alcohol exposure characteristically causes a decrease in the volume and neuronal density of the gray matter [32]. The affected areas extend over a broad region, including the parietal lobe, frontal lobe, hippocampus, amygdala, corpus callosum, basal ganglia, diencephalon, and cerebellum [32,33,34,35,36,37]. In the cerebellum, children with FASD show volume deficits in the anterior lobules related to sensorimotor functions (lobules I–V and VI) and lobules related to cognitive functions (crus II and lobule VIIB) [38]. Furthermore, the volumes of the cerebral and cerebellar white and gray matter are smaller than normal, and the extent of volume reduction correlates with clinical abnormalities in cognition and behavior [22].

### 2.2. Cognitive, Behavioral, and Motor Manifestations

FASD presents with a variety of cognitive and behavioral deficits. These are summarized in Table 1 based on the review by Mattson et al. [16,30]. Intellectual deficits are commonly observed findings, and prenatal alcohol exposure is generally associated with a low IQ score [16,30]. Attention deficits are also common and can be reliable markers for identifying children with prenatal alcohol exposure [16,30]. Children with FASD are less efficient and make more errors when processing visually presented information [39]. FASD children also show visual perception and construction impairments [40].

Executive functions are involved in goal-directed behaviors. These processes are impaired in children with FASD in a variety of domains [41], including speech tasks [42], response inhibition in a Go/NoGo task [43], problem-solving and planning [44], concept formation [44], and working memory [44]. Executive dysfunction is a core symptom in children who exhibit aggression and frequent delinquency during adolescence and adulthood [18]. Furthermore, children with FASD develop language deficits characterized by poor performance in word order, combining sentences, and grammatical comprehension [45]. 

Adaptive functioning, such as communication, socialization, and daily living skills, is also impaired in children with FASD [18,23,46].

Prenatal alcohol exposure is also associated with motor impairments [16,30]. The clinical features of motor impairments include defective fine motor control and balance [47]. Fine motor skills are more severely impaired than gross motor skills and can be implicated in actions requiring visual–motor control.

**Table 1:** Neurological symptoms of FASD.

Neurological Symptoms	Study Design	Ref.
Intellectual deficits	IQ comparison FASD: *n* = 41; mean age 13.7 (sd 3.47); mean IQ 91.6 (sd 14.37) Control: *n* = 46; mean age 13.3 (sd 3.64); mean IQ 110.0 (sd 12.09) FASD group exhibited significantly lower IQ.	[30]
Deficits in attention, especially visually presented information	Sustained attention was measured with “AK” subtests from a commercially available Continuous Performance Task program. FASD: *n* = 128; control: *n* = 53 Both groups: mean age 15.12 (sd 0.92) FASD group exhibited significantly more errors.	[39]
Deficits in visual perception and construction task	Visual hierarchical stimuli consisting of large (global) letters or shapes constructed from the arrangement of numerous smaller (local) letters or shapes FASD: *n* = 14; control: *n* = 14 Both group: age 9~16 years FASD subjects exhibited impairment in recalling local features relative to global features.	[40]
Deficits in executive functions, e.g., verbal fluency, response inhibition, problem-solving and planning, concept formation, and working memory	Neuropsychological test battery FASD: *n* = 10, age 13 years Control: *n* = 10, age 12 years 9 months FASD subjects exhibited greater difficulty than controls in tasks that involved the manipulation of information and goal management in working memory (e.g., planning, controlled oral word association, etc.).	[44]
Deficits in language, especially in word order, sentence combining, and grammatical comprehension	Formal communication skill assessments FASD: *n* = 8, age 4.5~9 years Control: *n* = 8, age 3.5~6 years FASD subjects exhibited mental age-inconsistent abilities in the comprehension and use of grammatical markers both in repetition and in spontaneous language tasks.	[45]
Deficits in adaptive functioning, e.g., communication, socialization, and daily living skills	Short Sensory Profile scores, Adaptive Behavior Assessment System Second Edition scores, and Wechsler Intelligence Scale Fourth Edition/Wechsler Preschool and Primary Scale of Intelligence Third Edition scores FASD and non-FASD: *n* = 46, age 3~14 years FASD subjects showed significantly lower scores on adaptative behavior than non-FASD subjects. No correlation was observed between IQ scores and adaptive behavior scores.	[46]
Deficits in fine motor controls and balance	Motor coordination test (balance; finger, hand, and foot coordination) Prospective longitudinal study of adults in two groups: adults previously diagnosed with FASD (*n* = 90) and adults who were exposed to varying levels of alcohol (*n* = 402). Only subjects who had been previously identified as having a diagnosis on FASD in childhood still showed deficits on motor tasks as adults.	[47]

This summary of symptoms is based on a review by Mattson et al. [14].

## 3. Role of the Cerebellum in Neurodevelopmental Disorders

There is a consensus that (a) cerebellar lesions that occur during early fetal life result in the dysfunction of cortical regions to which the affected cerebellum projects, and (b) such an impairment in the developing cerebellum leads to the altered development of the targeted cerebral regions and manifestations of permanent motor and behavioral symptoms [14,48]. This concept was formulated during the last two decades. Here, we review the notion that the cerebellum is involved in the manifestations of ASD and ADHD.

### 3.1. Closed and Reciprocal Cerebro-Cerebellar Circuits

The classic view by Allen and Tsukahara was that the cerebellum receives multiple inputs from various cortical regions and funnels this information back to M1 [49]. Strick and colleagues later challenged this concept based on experiments using neurotropic virus tracers [50]. The new concept indicates that cerebellar outputs influence broad regions of the cerebral cortex, including M1, premotor, prefrontal, and parietal areas [50]. Multiple and parallel closed-loop circuits connect the cerebellum with many cerebral cortical regions [50]. This architecture indicates a separate representation for the sensory–motor domain [the anterior lobe (lobules I–V) and lobule VIII] and the non-motor domains [posterolateral lobule VI and VII] in the cerebellum [14,51]. 

Coincidently, Schmahmann and colleagues described the cerebellar cognitive affective syndrome (CCAS, Schmahmann syndrome), which encompasses deficits in spatial cognition, executive function, and linguistic processing [11]. Patients with lesions in the posterolateral lobules show impairments in visual–spatial organization and memory (deficits in spatial cognition), abstract reasoning, planning, set-shifting, working memory (deficits in executive function), and agrammatism. The pathophysiology underlying these deficits is considered to be predictive impairment, which is widely seen in sensory–motor controls. Schmahmann used the notion of dysmetria of thought to stress the common pathophysiologies in sensory–motor and cognitive controls [11]. 

### 3.2. Functional Diaschisis and Sensitive Period

Wang et al. proposed that cerebellar dysfunction during childhood impacts the development of remote regions in the cerebral cortex, i.e., developmental diaschisis [52]. Diaschisis is defined as a sharp inhibition/reduction in activity at a site that is distant from a site of injury, but is anatomically connected with it through either direct or indirect fiber tracts [52]. The authors, citing the classic example of Hubel and Wiesel [53], emphasized that damage during a critical development period is irreversible. Accumulating evidence suggests that restricted lesions in the cerebellum during early life impair cerebral cortex functions, resulting in persistent cognitive symptoms. For example, damage to the cerebellar hemisphere in children causes a delayed acquisition of language and impaired visual and verbal reasoning, whereas damage to the vermis during childhood elicits withdrawn social behaviors, stereotyped behaviors, anxiety, and impaired gaze [54]. Lesions in the posterior fossa in children aged 6 to 13 sometimes induced cerebellar mutism, characterized by a regression of language capacities over time [55]. Some patients with cerebellar malformations develop ASD-like symptoms [56]. Furthermore, damage to the cerebellum during the prenatal period (premature birth or surgery) can be associated with social disability and high scores in ASD inventories [57,58]. 

These clinical observations suggest the existence of a critical period in the developing cerebro-cerebellar circuits, which was confirmed by designed experiments. For example, Gibson et al. [59] discussed the cerebellum’s role in ASD and emphasized the timing and critical periods of impaired cerebellar development that trigger ASD. The same group identified a critical period in a mouse model of tuberous sclerosis, using the mechanistic target of the inhibitor rapamycin to pharmacologically inhibit dysregulated downstream signaling. Furthermore, the mechanism of the critical period has been studied from the perspective of circuit development. Cerebellar development starts at an early stage during embryogenesis and extends to after delivery. There is a major increase in volume in the third trimester of pregnancy. This trimester is considered the critical period for cerebellar maturation [60]. A positive relationship was found between nutrition and white matter maturation [61]. In this regard, disruption in the third trimester of pregnancy might impact the maturation of functional connectivity. On the other hand, Hoffman et al. [62] identified the cellular mechanisms underlying the onset and offset vulnerability. They described a critical period for neuroinflammation in relationship to gene expression with a strict temporal profile in PCs [62]. 

It should be noted that the cognitive symptoms documented in these studies are permanent. In contrast, the motor symptoms induced by acquired lesions during childhood often wane with time [63]. This discrepancy suggests that the vulnerability to perturbation depends on the motor and cognitive domains [52]. In agreement with this assumption, imaging studies have shown sensory–motor functional connectivity between the cerebral cortex and the cerebellum in infants, compared with functional connectivity between executive control and default mode systems in children and adults [64]. 

### 3.3. Autism Spectrum Disorder and Attention-Deficit-Hyperactivity Disorder as Neurodevelopmental Disorders

While the underlying mechanisms of ASD are complex [48], various structural and functional abnormalities have been described in the cerebellum of ASD patients. Studies using voxel-based morphometry (VBM) showed significant structural changes in the posterior lobule VI and crura I and II (both parts of lobule VII) in these patients [65,66]. Furthermore, Limperopoulos and colleagues [67] reported the presence of cerebellar lesions in the perinatal period and that such lesions tended to reduce the volume of the cerebellar gray matter and remote neocortical regions. In addition, functional MRI (fMRI) studies showed an overall decrease in functional connectivity between the cerebral cortex and the cerebellum [68]. Other fMRI studies in patients with ASD have also shown an atypical activation of the cerebro-cerebellar networks during tasks of motion perception and social interaction perception [69]. 

Structural and functional abnormalities have also been described in the cerebella of patients with ADHD, which are characterized clinically as inattention, hyperactivity, and impulsivity [65,66]. The severity of the clinical features correlates with the degree of volume reduction in the posterior vermis [70]. Other groups showed a bilateral decrease in the gray matter in lobule IX [65,66]. Importantly, methylphenidate, a drug used in ADHD, augments the activation of fronto-striato-cerebellar regions, thereby normalizing the connectivity in these regions and normalizes the connectivity in children with ADHD [71]. 

**Figure 2:**
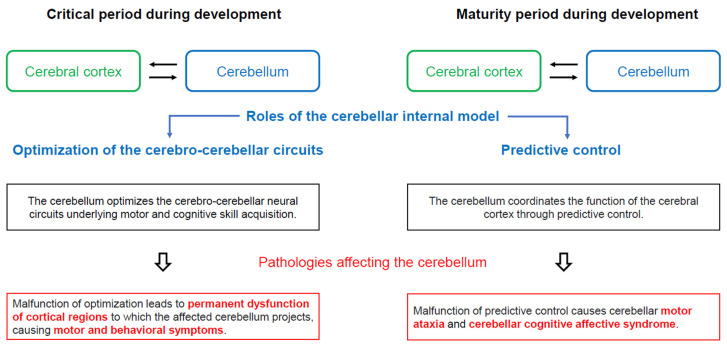
Assumed physiological significance of closed cerebro-cerebellar loop. In the critical period during development (mainly during postnatal period or childhood), the cerebellar internal model optimizes the cerebro-cerebellar neural circuits involved in motor and cognitive skill acquisition. Thus, cerebellar dysfunction during the critical period leads to permanent dysfunction of the cortical regions to which the affected cerebellum projects, with the associated motor and behavioral symptoms. In contrast, during the maturity period of development, the cerebellum optimizes the function of the cerebral cortex through predictive controls. Thus, cerebellar lesions manifest clinically as motor ataxias and cerebellar cognitive affective syndrome (CCAS/Schmahmann syndrome), which encompass deficits in spatial cognition, executive function, and linguistic processing.

When considered together, the cerebellum seems to play a critical role in the functional maturation of the cerebro-cerebellar circuits underlying skill acquisition [65,66]. In this regard, Cundari et al. [66] introduced the concept of “optimization of cerebello-cerebral circuits” to explain the role of the cerebellum (Figure 2). Such optimization seems to depend on a schedule unique to the motor and cognitive domains. Consequently, malfunction of the connectivity between the cerebral cortex and the cerebellum could be one of the pathophysiological mechanisms of various neurodevelopmental disorders, such as ASD and ADHD. 

### 3.4. The Internal Model within the Cerebellum

This section explores the concept derived from clinical observations, commonly called the optimization of cerebello-cerebral circuits. Recent computational theories on cerebellar function guided our approach. 

**Figure 3:**
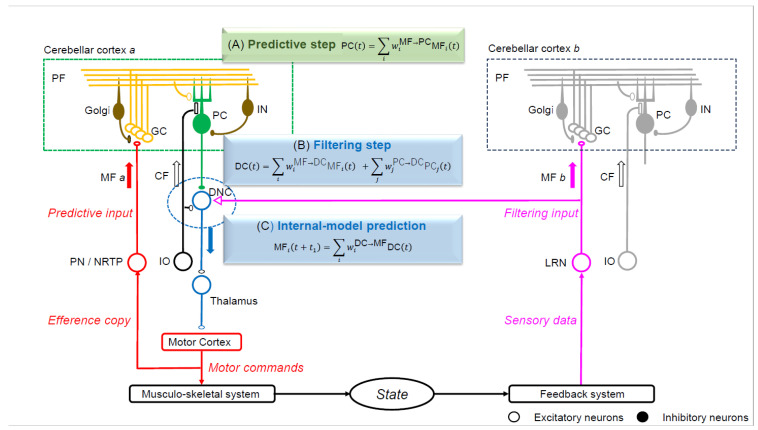
The Kalman filter model that predicts the motor cortex activity. The linear equations of neuron activities resemble those of an estimator known as the Kalman filter. The current status is transformed into a predictive state in the cerebellar cortex (prediction step), whereas the predictive state and sensory feedback from the periphery are integrated into a filtered state at the dentate nucleus (filtering step). One mossy fiber (MF) input (MF a) from the pontine nuclei (PN) or nucleus reticularis tegmenti ponitis (NRTP) projects to the cerebellar cortex (cerebellar cortex a) without collateral projection to DNCs. The MF a input to cerebellar cortex a generates a prediction input to dentate nucleus cells (DNCs). Another MF input (MF b) from the lateral reticular nucleus (LRN) projects to the cerebellar cortex (cerebellar cortex b) with a collateral to DNCs, which conveys sensory feedback information (filtering input). MF a and MF b have distinct sources and provide prediction and filtering inputs to DNCs, respectively. PF, parallel fiber; Golgi, Golgi cell; GC, granule cell; PC, Purkinje cell; IN, interneuron; MF, mossy fiber; CF, climbing fiber; DNC, dentate nucleus cell; IO, inferior olive nucleus; PN, pontine nucleus; NRTP, nucleus reticularis tegmenti pontis; LRN, nucleus reticularis tegmenti pontis; INPC, interpositus nucleus cell.

The internal forward model transforms a motor command into a prediction of its outcome regarding the sensory reafference the movement will cause [72,73]. A consensus exists that such an internal forward model embedded within the cerebellum acts as a neural substrate for predictive motor control [74,75]. Our proposal posits that the cerebellar internal forward model is conceptualized as a Kalman filter model, comprising two essential steps (Figure 3) [75,76]: 

(a) Prediction step: The prediction state is computed based on the current state.

(b) Filtering step: The predictive state is integrated with sensory feedback. 

The PCs compute the predictive state within the cerebellar cortex using information conveyed by mossy fibers (MFs). Simultaneously, the dentate nucleus cells (DNCs) combine the predicted state from PCs with sensory feedback originating from the MFs [75,76]. 

Furthermore, facilitated by the internal forward model, the predictive control drives motor learning toward further improvements [77]. Sensory prediction errors play a crucial role in driving adaptive changes during visuomotor tasks [78]. Through the continuous accumulation of such predictive control, the learning process becomes feasible. Such a capability for internal model reconstruction also serves as the capacity for compensation and restoration in response to pathologies, a phenomenon described as the cerebellar reserve [79].

We propose that internal models structured around a Kalman filter are not only involved in the predictive control of motor tasks and learning but also play a significant role in the functional connectivity with various brain regions during the developmental phase. The Universal Cerebellar Transform Theory, which postulates that the operational principles of the cerebellum extend across both the motor and cognitive domains [80], suggests that the cerebellar internal model may be functionally connected to the motor and cognitive areas of the cerebral cortex through the same mechanism.

### 3.5. Neural Development of the Cerebellar Cortex and Failure of the Internal Model

**Figure 4:**
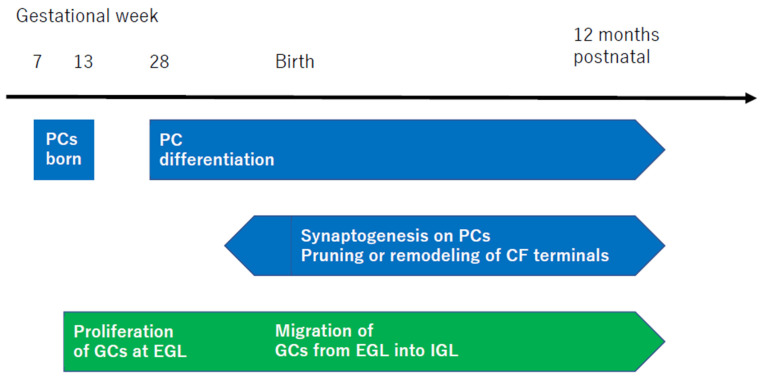
Schematic representation of the development of cerebellar cells and synapses. The time of development is cited from [48]. The 28th week of human gestation corresponds to P0 in mouse cerebellar development. PC, Purkinje cell; CF, climbing fiber; GCs, granule cells; EGL, external granule layer; IGL, inner granule layer.

The vulnerability of the internal model with a Kalman filter structure to disorders during fetal development is evident from the formation of unique neural circuits in the cerebellum. The development of the human cerebellum characteristically continues for a longer period than that of the cerebrum, extending until one year after birth [81] (Figure 4). 

PCs first appear in humans during gestational weeks 7–13, followed by extensive complexity in both dendritic length and arborization at gestational week 28 [48]. Conversely, granule cells (GCs) initially proliferate in the external granule layer (EGL) at gestational week 10, continuing until the first few postnatal months, peaking at the fourth postnatal month [48]. This is followed by the migration of GCs into the inner granule layer (IGL) [48]. From the prenatal period just before birth to one year after birth, there is an active phase of further synaptogenesis. This process includes the pruning of CFs [82], the remodeling of CF terminals into a mature configuration [83], and the extension of axons of PCs to DNCs [84]. 

Thus, the initial proliferation of PCs and the EGL during the fetal period is essential for the “foundational elements of the internal model with a Kalman filter structure”, with the critical period extending to up to one year after birth.

In conclusion, the dysregulation of the “optimization of the cerebello-cerebral circuits” may be attributed to a failure of “optimization between the cerebellar internal model and cerebral regions” (Figure 2).

## 4. Malfunctioning Connectivity between the Cerebral Cortex and Cerebellum in FASD

Prenatal exposure to alcohol can cause structural and functional abnormalities in the cerebral cortex or subcortex, resulting in a variety of permanent cognitive and motor deficits in FASD. In addition, accumulating evidence suggests that alcohol exposure damages the developing cerebellum and, consequently, disorganizes the functional connectivity of the cerebro-cerebellar neural circuits, leading to a worsening of the primary cortex dysfunctions in FASD. We stress the following evidence:

(a) Similarities in cognition symptoms between ADHD and FASD: Mattson et al. [16] have underlined the similarities between FASD and ADHD. Both groups of children show common deficits in attention, executive dysfunctions, lack of response inhibition, and deficits in adaptivity [16,24,85].

(b) Possible impairment of functional connectivity: Children with FASD show an impairment in cognitive flexibility, which requires coordination among various cortical and subcortical regions [22]. Consistent with this notion, Diffusion Tensor Imaging (DTI) tractography has accentuated the inter-hemispheric white matter abnormality [17]. Functional imaging in children with FASD also shows altered activation patterns in tasks of visual attention [86], verbal learning [34], response inhibition [87], and working memory [88]. Children with FASD also show volume deficits in the anterior lobules related to sensorimotor functions (lobules I, II, IV, V, and VI), and lobules related to cognitive functions (crus II and lobule VIIB) [38]. These findings suggest a possible malfunction of the cerebro-cerebellar networks. In addition, the impairment in synaptic plasticity observed in FASD [26] may be related to the inability to organize neural circuits properly.

## 5. TOMCCLs: Therapies Oriented towards the Malfunctioning of the Cerebello-Cerebral Loops 

Therapies targeting the cerebello-thalamo-cortical tract have not yet been developed. We discuss here potential therapies aiming to counteract the malfunctioning of the cerebello-cerebral loops in the context of alcohol toxicity.

It is believed that there is no effective therapy for FASD [89]. One of the utilized therapeutic targets is alcohol-induced neuroinflammation, which significantly contributes to the early postnatal mouse model [89]. It was shown that neuroinflammation persists in the days following cessation of ethanol administration, impairing the microglial ramifications around the PCs, reducing the expression of IL-23a and IL-12Rbeta1, and reducing the expression of CX3CL1. IL-23 plays important roles in the activation of Th17-mediated autoimmunity, whereas CX3CL1 serves as a regulator of microglia activation. Kane and colleagues showed that ethanol impacts the pathways critical for immune responses. These findings suggest that the therapeutic effects of inhibiting proinflammatory molecules need to be tested experimentally. The selective activation of microglia near PCs could be evaluated to test hypotheses surrounding inflammation-induced PC death [89]. CBI (cerebellum–brain inhibition) could be used to assess the efficacy of these anti-inflammatory therapies on improving the function of cerebello-cerebral projections.

**Figure 5:**
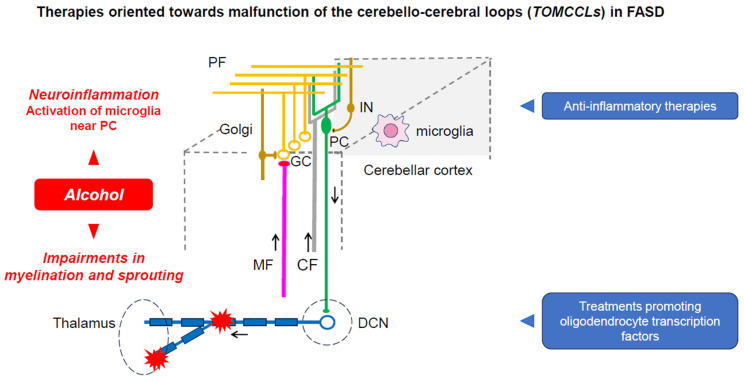
Therapies blocking neuroinflammation and promoting myelination/sprouting within the cerebellar circuitry block the effects of ethanol on the cerebellar circuitry, restoring the capacity of the cerebellar circuitry to generate and maintain internal models.

Another potential approach is the modulation of cerebellar myelination. Niedzwiedz-Massey and colleagues have studied the effects of alcohol on oligodendrocyte lineage cells [90]. Alcohol significantly reduces the expression of markers for the multiple stages of maturation, impairs the expression of molecules regulating the differentiation of oligodendrocytes, and reduces the expression of myelin-associated inhibitors known to compensate for alcohol toxicity. Myelin-associated inhibitors regulate axonal sprouting and myelination especially through a regulation of the Nogo receptor complex. It is likely that alcohol’s effect on myelin represents a potential therapeutical target, as white matter abnormalities are common in FASD and they persist in adults [17,91]. The expression of transcription factors critical for the differentiation of oligodendrocytes (Olig1, Olig2, Sox-10, NKX2.2, MRF) are all reduced by alcohol. It is necessary to assess novel drugs that aim to counteract these effects. A recent study using a rat model demonstrated that the correction of thiamine deficiency led to the restoration of these transcription factors [92]. The impairment of myelin impacts the propagation of action potentials and therefore therapeutical approaches targeting the myelination process could be evaluated by electrophysiological techniques. In other words, the therapeutic principle underlying TOMCCLs is based on the perspective of the Kalman filter model, restoring the predictive step in the cerebellar cortex and filtering step in the dentate nucleus (Figure 5). 

## 6. Conclusions

The presented perspective outlines the necessity to clarify the reality of optimization of cerebro-cerebellar circuits at the neural circuit level for the treatment of FASD. It is emphasized that addressing the foundational elements of the internal model might offer valuable clues. Identifying the critical period for optimization of cerebro-cerebellar networks could open the door to therapeutic intervention. Both the neuroinflammation pathways and oligodendrocyte differentiation represent attractive options for treating FASD symptoms. We speculate that TOMCCLs restore the cerebellar cortex’s and cerebellar nuclei’s physiological activities, promoting the cerebello-cerebral interactions that underlie motor, cognitive, and social skills. Our approach based on cerebello-cerebral interactions highlights the importance of the cerebellum as a hub during development.

## Data Availability

The concept reported in this manuscript is not associated with any raw data.
